# A worldwide bibliometric analysis of malignant peripheral nerve sheath tumors from 2000 to 2022

**DOI:** 10.3389/fonc.2023.1111985

**Published:** 2023-01-27

**Authors:** Xingfeng Huang, Zexin Fu, Qinhao Gu, Ji Wang, Yi Sun, Yong He, Sufan Wu, Xiaojie Hu, Chengrui Guo

**Affiliations:** ^1^ Center for Plastic & Reconstructive Surgery, Department of Plastic and Reconstructive Surgery, Zhejiang Provincial People’s Hospital (Affiliated People’s Hospital, Hangzhou Medical College), Hangzhou, Zhejiang, China; ^2^ Cancer Center, Zhejiang University, Hangzhou, China; ^3^ State Key Laboratory of Fluid Power and Mechatronics Systems, School of Mechanical Engineering, Zhejiang University, Hangzhou, China; ^4^ Key Laboratory of Materials Processing and Mold, Zhejiang University, Zhengzhou, China; ^5^ Key Laboratory of 3D Printing Process and Equipment of Zhejiang Province, College of Mechanical Engineering, Zhejiang University, Hangzhou, China; ^6^ Department of Plastic and Reconstructive Surgery, Shanghai Ninth People’s Hospital, Shanghai Jiao Tong University School of Medicine, Shanghai, China

**Keywords:** bibliometric analysis, malignant peripheral nerve sheath tumors, cancer, VOSviewer, RStudio

## Abstract

**Background:**

Currently, malignant peripheral nerve sheath tumors (MPNST) are the subject of intense research interest. However, bibliometric studies have not been conducted in this field. The purpose of the study was to identify historical trends and presents a bibliometric analysis of the MPNST literature from 2000 to 2022.

**Methods:**

For the bibliometric analysis, publications were retrieved from the Web of Science database based on the following search terms: [TI = (MPNST) OR TI= (malignant peripheral nerve sheath tumors) AND PY = (2000–2022)]. The following information was collected for each document: the publication trends and geographical distribution, important authors and collaboration, keyword distribution and evaluation, most popular journals, and most influential articles.

**Results:**

We included 1400 documents for bibliometric analysis, covering five categories: 824 articles, 17 proceedings papers, 68 letters, 402 meeting abstracts, and 89 reviews. Corrections, editorials, book chapters, data papers, publications with expressed concerns, and retractions were excluded from our research.

**Conclusion:**

Since 2000, the number of publications on MPNST has continuously increased. Among all countries that contributed to the MPNST research, the USA, Japan, and China were the three most productive countries. The journal *Modern Pathology* has the most publications on MPNST, while those in the *Cancer Research* journal were the most frequently cited. The University of Texas MD Anderson Cancer Center may be a good partner to collaborate with. Recent research trends in MPNST have focused on tumorigenesis, clinical management, and predictive biomarkers.

## 1 Introduction

Malignant peripheral nerve sheath tumors (MPNST) are highly aggressive soft-tissue sarcomas originating from peripheral nerves (1). Approximately 50% of patients with MPNST also have neurofibromatosis type 1 (NF1), often due to malignant transformation from plexiform neurofibroma (2, 3). Like most sarcomas, MPNST has a high probability of local recurrence and metastatic spread. Unfortunately, no targeted therapies have proven effective to date, resulting in a relatively poor prognosis (4).

Previous studies found that deletion of the *CDKN2A* gene is the most common secondary mutation based on *NF1* loss, followed by *TP53* mutation (3, 5). In addition, *PRC2, SUZ12*, and *EED* mutation is also frequently observed in MPNST, and loss of *H3K27me3* contributes to MPNST progression (6). These clues reveal the potential treatment of BRD4 and BET inhibition in MPNST (7).

Many researchers have provided new insights into MPNST since the onset of the 20th century. Yearly, many articles are published about MPNST, exploring and demonstrating clinical management, the tumorigenesis mechanism, and candidate molecular targets. However, no bibliometric analyses of MPNST-related studies have been published to date. It is vital for researchers and surgeons to be aware of the qualitative and quantitative characteristics of influential research in MPNST so that they can contextualize their clinical practices and aid in shaping future research. As a result, we conducted a bibliometric analysis of the MPNST literature to provide narrative synthesis and evaluate research trends.

## 2 Materials and methods

### 2.1 Database and search strategy

Bibliometric analysis on MPNST was conducted using Web of Science (WOS) core data as of August 2022.

Research articles on MPNST in any language were identified by using the search terms [TI = (Malignant peripheral nerve sheath tumor OR MPNST) AND PY = (2000–2022)]. As part of our search optimization process, we checked that the title of every article was relevant to the research.

**Figure 1:**
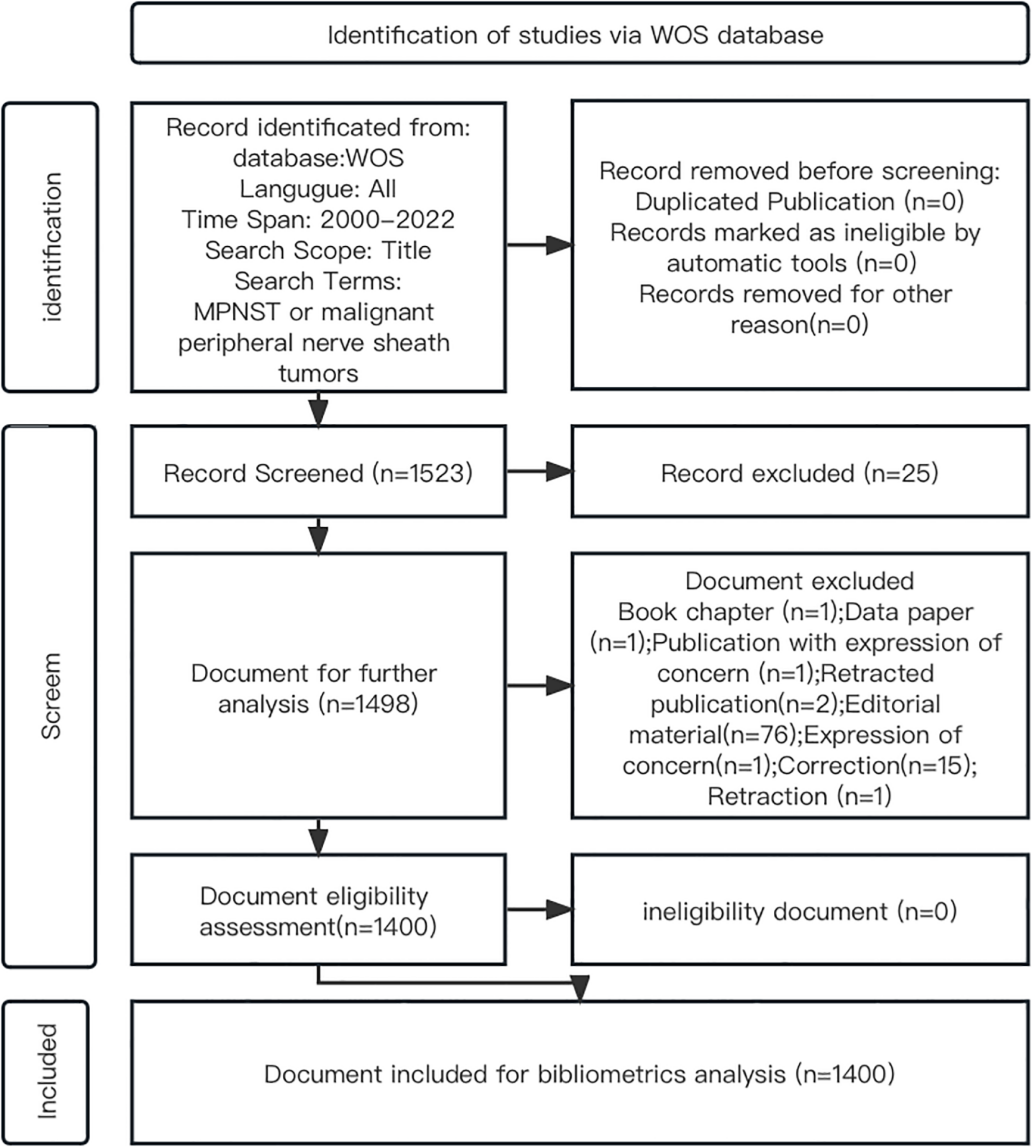
A flowchart showing the search strategy.

The data were downloaded and analyzed separately by two researchers, and in the event of any discrepancies, a third independent researcher was invited to reproduce and analyze the data and make the final decision with all authors (**Figure 1**).

### 2.2 Information extraction

After two researchers had reviewed the retrieved data independently, bibliometric analysis was conducted on all the retrieved documents using four different software packages:

1) A spreadsheet using Microsoft Excel 2019 was used to calculate the percentages and frequencies of published materials.
2) A visual analysis of the bibliometric networks was compiled using VOSviewer (version 1.6.18).
3) Citation metrics and the temporal distribution of the publications were determined using RStudio.
4) A keyword citation burst analysis was performed on CiteSpace.

### 2.3 Bibliometric indices

The H-index indicates that an author has published at least h papers relevant to the MPNST that have each been cited at least h times. The G-index gives more weight to highly cited articles, as the top G articles receive at least G^2^ citations. The M-index is calculated by dividing a researcher’s H-index by their academic age (years since the first publication).

## 3 Results

### 3.1 Overview of retrieved literature

A total of 1498 articles were identified from the WOS database, excluding those not written in English (25 articles). The composition of the retrieved literature based on document type is summarized in **Table 1**.

**Table 1:** Categories of publications in MPNST In the period 2000–2022.

Document type	Total publications (TP)	Percentage (%)
article	824	55.01
book chapter	1	0.07
data paper	1	0.07
proceedings paper	17	1.13
publication with expression of concern	1	0.07
retracted publication	2	0.13
correction	15	1.00
editorial material	76	5.07
expression of concern	1	0.07
letter	68	4.54
meeting abstract	402	26.84
retraction	1	0.07
review	89	5.94
Total	1498	100

Among all the documents, articles (824, 55.1%) topped the list, followed by meeting abstracts (402, 26.84%). Others include review articles (89, 5.94%), letters (68, 4.54%), and proceedings paper (17,1.13%). Corrections, editorials, book chapters, data papers, publications with expressed concerns, and retractions were excluded from our research. Five document types are included: articles, reviews, meeting abstracts, conference proceedings, and letters. Consequently, 1400 articles met the criteria for inclusion.

### 3.2 Citation and publication trends over time

**Table 2** presents statistics for MPNST research publications. Among the most productive years, 2018 had 104 documents, while 2000 (excluding 2022) only had 20 documents. Between 2002 and 2003, the number of publications grew at the fastest rate. A significant decline in article productivity was evident between 2014 and 2015. Article production in 2015 was more than 30% lower than in 2014. The number of published articles in the investigated period fluctuated, but overall, increased in trend. **Table 2** shows the annual scientific publications and citations. Among the total citations, 2014 had the highest number with 1516, followed by 2002 with 1424. It is reasonable that recent articles, such as those published in 2022 and 2021, received fewer citations. According to a global review of the literature from the last two decades, the number of citations per article peaked in 2002 with an average of 67.81 citations per article.

**Table 2:** Citations and number of articles yearly.

Year	Actual value			Normalized value		
	Articles	Average citation	Citations	Articles	Average citation	Citations
2000	20	26.60	532	0.19	0.39	0.35
2001	23	32.13	739	0.22	0.47	0.49
2002	21	67.81	1424	0.20	1.00	0.94
2003	30	26.93	808	0.29	0.40	0.53
2004	36	20.36	733	0.35	0.30	0.48
2005	44	25.43	1119	0.42	0.38	0.74
2006	45	24.84	1118	0.43	0.37	0.74
2007	41	14.85	609	0.39	0.22	0.40
2008	54	22.52	1216	0.52	0.33	0.80
2009	62	19.77	1226	0.60	0.29	0.81
2010	64	13.86	887	0.62	0.20	0.59
2011	60	14.87	892	0.58	0.22	0.59
2012	83	11.28	936	0.80	0.17	0.62
2013	75	15.91	1193	0.72	0.23	0.79
2014	93	16.30	1516	0.89	0.24	1.00
2015	67	8.82	591	0.64	0.13	0.39
2016	73	15.70	1146	0.70	0.23	0.76
2017	93	9.13	849	0.89	0.13	0.56
2018	104	3.63	378	1.00	0.05	0.25
2019	94	4.82	453	0.90	0.07	0.30
2020	100	2.60	260	0.96	0.04	0.17
2021	103	1.12	115	0.99	0.02	0.08
2022	7	0.14	1	0.07	0.00	0.00

### 3.3 Most influential authors

**Figure 2:**
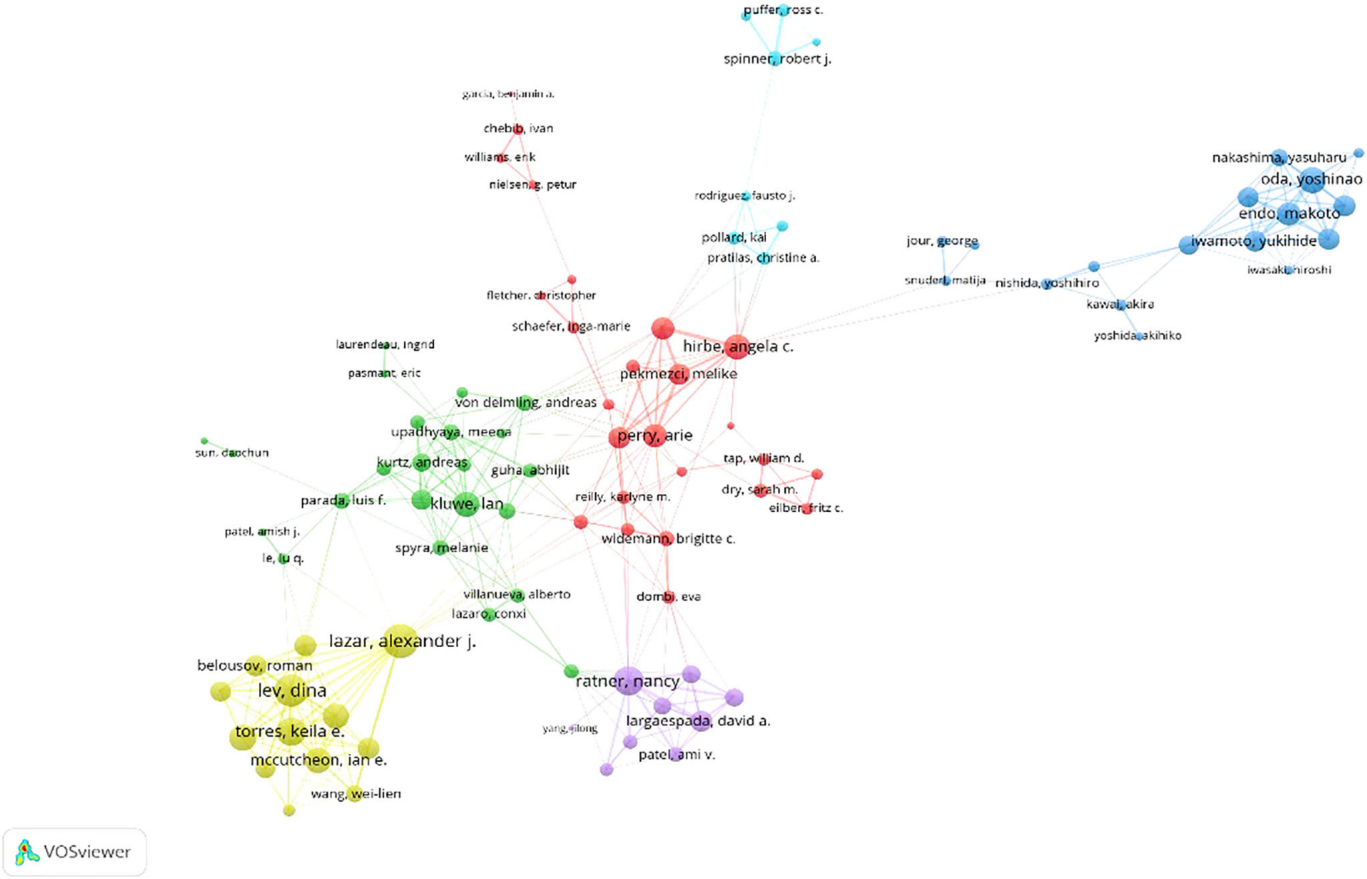
An overview of the co-authorship network in MPNST research VOSviewer.

The MPNST research involved 6000 authors. It is interesting to note that only 19 publications have been written by a single author. Among the documents, 19 were single-authored publications, whereas the rest were multi-authored. Each article had 6.51 co-authors on average. As a result, 98.6% of MPNST researchers collaborated during their research. Based on publications and citations since 2000, the top 10 authors are listed in **Table 3**. Victor F Mautner (University Medical Center Hamburg-Eppendorf) tops the list with 38 articles, followed by Steven L. Carroll (Medical University of South Carolina) with 32 documents. Mautner’s H, G, and M indices were respectively 21, 30, and 1.05. The top three most cited authors were David H Gutmann (1121), Victor F Mautner (1090), and Christopher DM Fletcher (1059). From 2000–2008, Washington University dominated the MPNST research field, whereas the University of Texas MD Anderson Cancer Center has held the top position since 2009. VOSviewer was used to visualize authors, with a minimum of five documents and ten citations needed for an author to be considered (**Figure 2**). MPNST research was impacted by the teams of Arie Perry, Nancy Ratner, Melike Pekmezci, and Alexander J. Lazar.

**Table 3:** The top 10 authors ranked by publications and citations.

Rank	Author	Publication	Country	Institution	H-index	G-index	M- index	TC	PY Start
1	Victor F. Mautner	38	GERMANY	University Medical Center Hamburg-Eppendorf	21	30	1.05	1090	2003
2	Steven L Carroll	32	USA	Medical University of South Carolina	11	19	0.55	371	2003
3	Arie Perry	27	USA	University of California San Francisco	12	13	0.545	867	2001
4	Nancy Ratner	27	USA	Cincinnati Children’s Hospital Medical Center	14	21	0.824	815	2006
5	Melike Pekmezci	23	USA	University of California San Francisco	5	8	0.5	193	2013
6	Alexander J. Lazar	19	USA	University of Texas M.D. Anderson Cancer Center	12	17	0.857	899	2009
7	Andreas von Deimling	19	GERMANY	University of Heidelberg	13	13	0.65	674	2003
8	Lan Kluwe	18	GERMANY	University Medical Center Hamburg-Eppendorf	13	14	0.684	701	2004
9	Ragnhild A. Lothe	17	NORWAY	University of Oslo	11	13	0.5	497	2001
10	Fredrik Mertens	17	SWEDEN	Lund University	9	13	0.409	459	2001

### 3.4 Publications and citations across geographic regions

**Figure 3:**
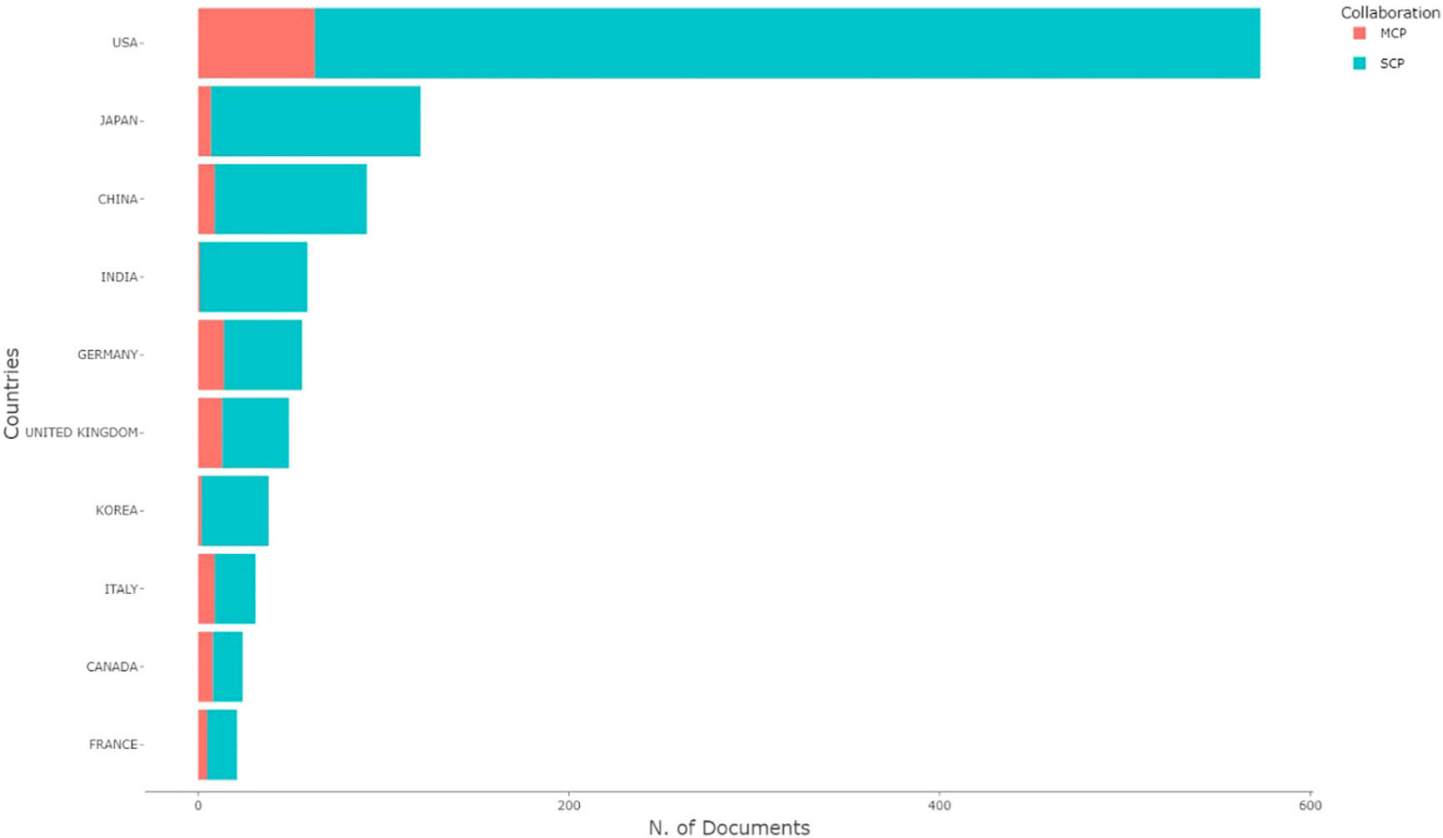
Publications with single and multiple country authorship.

**Figure 4:**
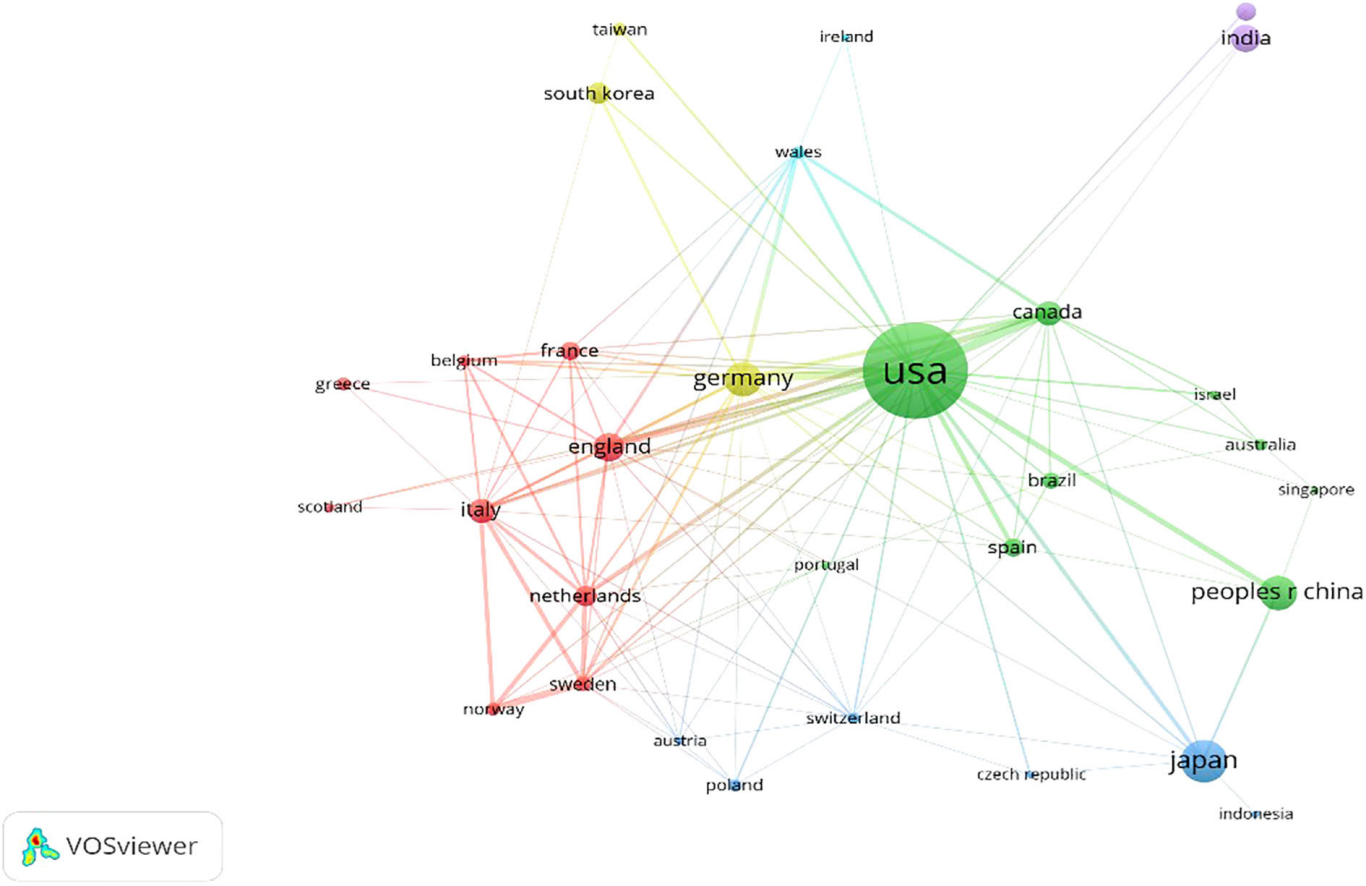
International collaboration network map. The widths of the connection lines indicate the collaboration strength. Countries with similar colors form a cluster VOSviewer.

Contributions to the field of MPNST were made by scholars from 49 countries. As illustrated in **Table 4**, the top 10 countries with high production are listed. The USA ranked 1 with 573 articles, while Japan ranked second with 120. **Figure 3** presents the distribution of articles wrote by single and multiple countries. Authors from several countries, such as Finland, New Zealand, Lebanon, and Slovenia, published articles cooperating with authors from multiple countries. In the most cited countries list, the USA gained the top spot with 8764 citations, followed by the United Kingdom with 2184 citations. On the other hand, considering citations for each article, the United Kingdom ranks first with 44.57 citations per article, and Norway is second with 33.67 citations per article. In **Figure 4**, the cooperation network between the countries is mapped. As one of the most important contributors to MPNST research, the USA has relatively frequent collaborations with China, Germany, Canada, and Japan. Despite India being ranked fifth in total citations, cooperation with other countries did not occur frequently.

**Table 4:** The top 10 countries based on publications and citations.

Rank By publications	Country	Articles	Citations	Average citations (AC)	Percentage (%)	SCP	MCP	Rank by AC
1	USA	573	8764	15.29	54.78	510	63	6
2	JAPAN	120	1449	12.07	12.14	113	7	7
3	CHINA	91	682	7.49	8.81	82	9	8
4	INDIA	59	167	2.83	6.23	58	1	10
5	GERMANY	56	1473	26.3	4.51	42	14	4
6	UNITED KINGDOM	49	2184	44.57	3.87	36	13	1
7	KOREA	38	207	5.45	3.87	36	2	9
8	ITALY	31	1010	32.58	2.36	22	9	2
9	CANADA	24	402	16.75	1.72	16	8	5
10	FRANCE	21	573	27.29	1.72	16	5	3

SCP, single country publications; MCP, multiple country publications.

### 3.5 Keyword analysis

#### 3.5.1 Keywords hotspot

Keywords were calculated using RStudio from 1400 retrieved documents, resulting in 1509 author keywords (DE) and 1519 author keywords-plus (ID). The most frequently used author keywords (DE) were: “malignant peripheral nerve sheath tumor”, “MPNST”, “neurofibromatosis type 1”, “sarcoma”, “nf1”, “schwannoma”, “immunohistochemistry”, “chemotherapy”, “MRI”, and “radiotherapy”. The following hot keywords were included in the author keywords-plus (ID) group: “schwannoma”, “expression”, “survival”, “neurofibromatosis type 1”, “cancer”, “soft-tissue sarcomas”, “Schwann-cells”, “benign”, “gene”, and “management”. The frequency results are presented in **Table 5**.

**Table 5:** Distribution of keywords based on frequency.

Rank	Author keywords (DE)	Frequency	Keywords-plus (ID)	Frequency
1	malignant peripheral nerve sheath tumor	404	schwannoma	69
2	MPNST	139	expression	68
3	neurofibromatosis type 1	68	survival	62
4	sarcoma	58	neurofibromatosis type 1	59
5	nf1	48	cancer	54
6	schwannoma	35	soft-tissue sarcomas	69
7	immunohistochemistry	31	Schwann-cells	68
8	chemotherapy	19	benign	62
9	MRI	17	gene	59
10	radiotherapy	15	management	54

#### 3.5.2 Subdisciplines

**Figure 5:**
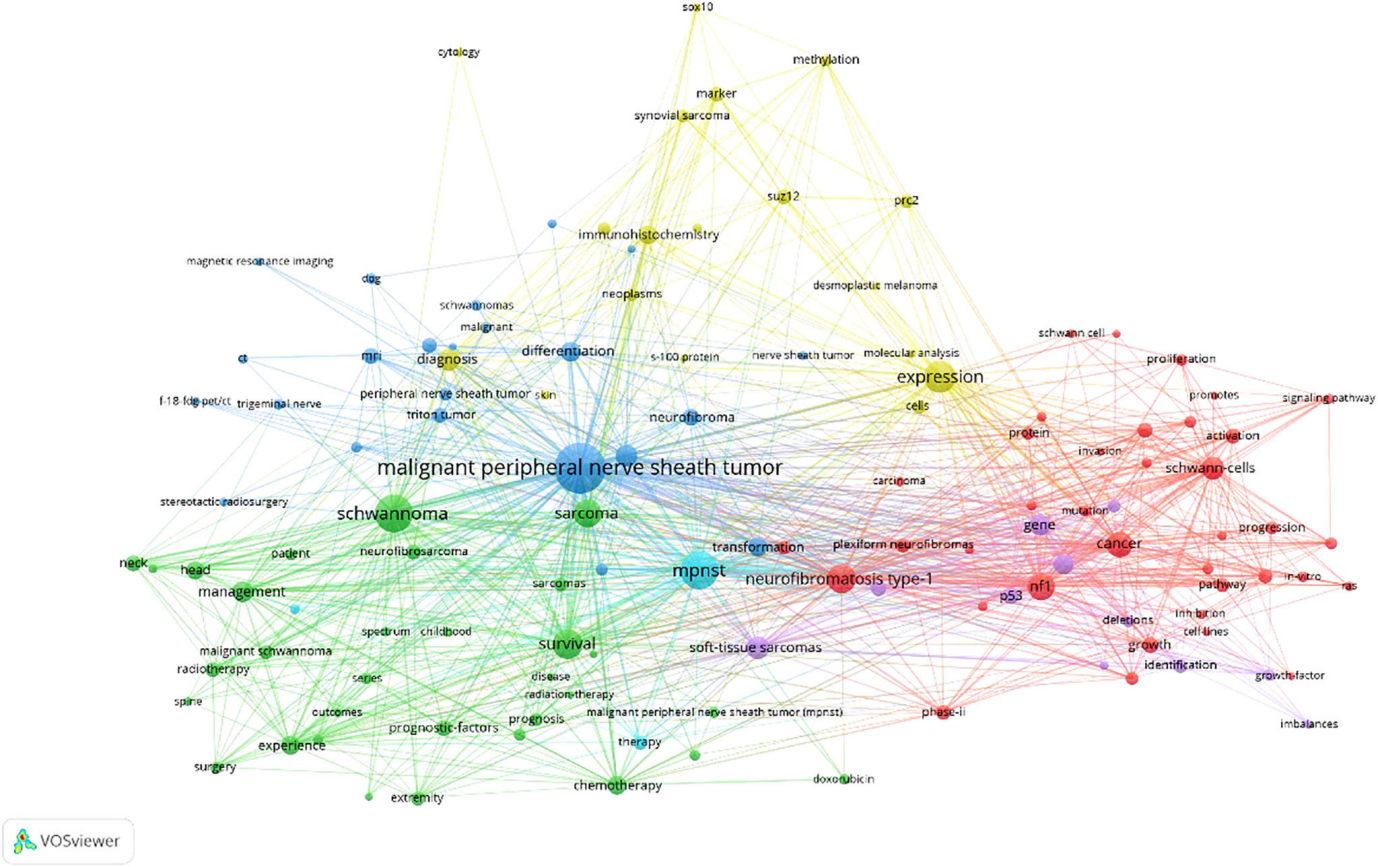
The authors’ keywords are visualized in a network map. Keywords with similar colors form a cluster VOSviewer.

**Figure 6:**
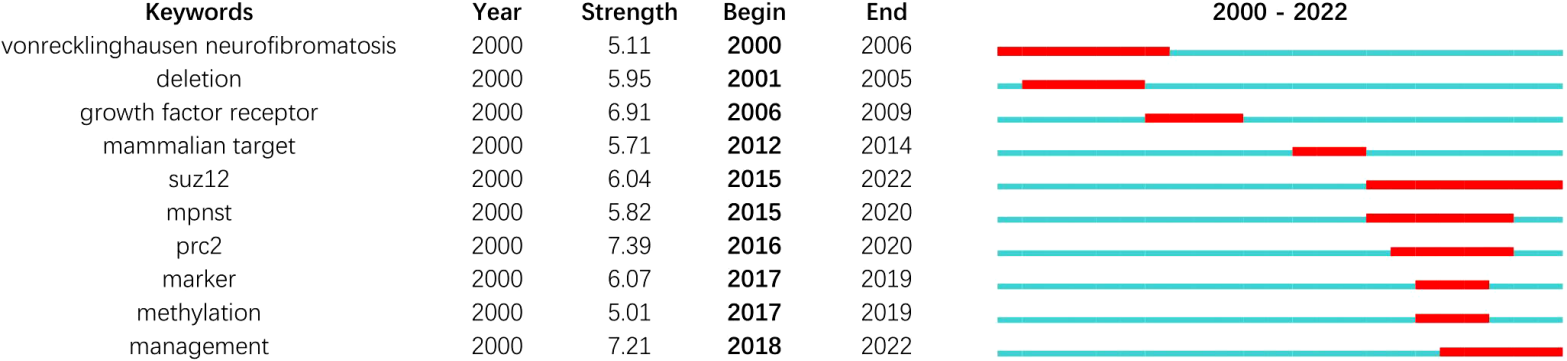
Citation bursts for the top 10 keywords.

As displayed in **Figure 5**, “malignant peripheral nerve sheath tumor”, “MPNST”, and “Schwannoma” are the author keywords that appear most frequently. A cluster analysis was conducted on keywords that met the selection criteria. Circles within the same color clusters indicate publications with similar topics. Specifically, the cluster of keywords in red (cluster 1,37) associated with the topic “neurofibromatosis type 1” includes the following: “Activation”, “Angiogenesis”, “Growth factor”, “Invasion”, “Mutation”, “Plexiform Neurofibroma”, “*NF1* gene”, “Proliferation”, and “Ras”. The cluster of keywords in green (cluster 2,31) associated with the topic “Schwannoma” includes the following: “Chemotherapy”, “Doxorubicin”, “Head”, “Radiotherapy”, “Sarcoma”, “Surgery”, “Survival”. The blue cluster (cluster 3,23) included the keywords “Differentiation”, “F-18-fdg PET/CT”, “MRI”, “Neurofibroma”, “Trigeminal Nerve”, and “Triton Tumor” associated with the topic “malignant peripheral nerve sheath tumor”. Keywords in the yellow cluster (cluster 4,18) such as “Methylation”, “*PRC2*”, “S-100 Protein”, “*SOX10*”, and “*SUZ12*” were associated with the topic “Expression”. The top 10 keywords for the last two decades can be seen in **Figure 6**. Recently, the keyword “*PRC2*” has been the most cited.

### 3.6 Preferred journals

**Figure 7:**
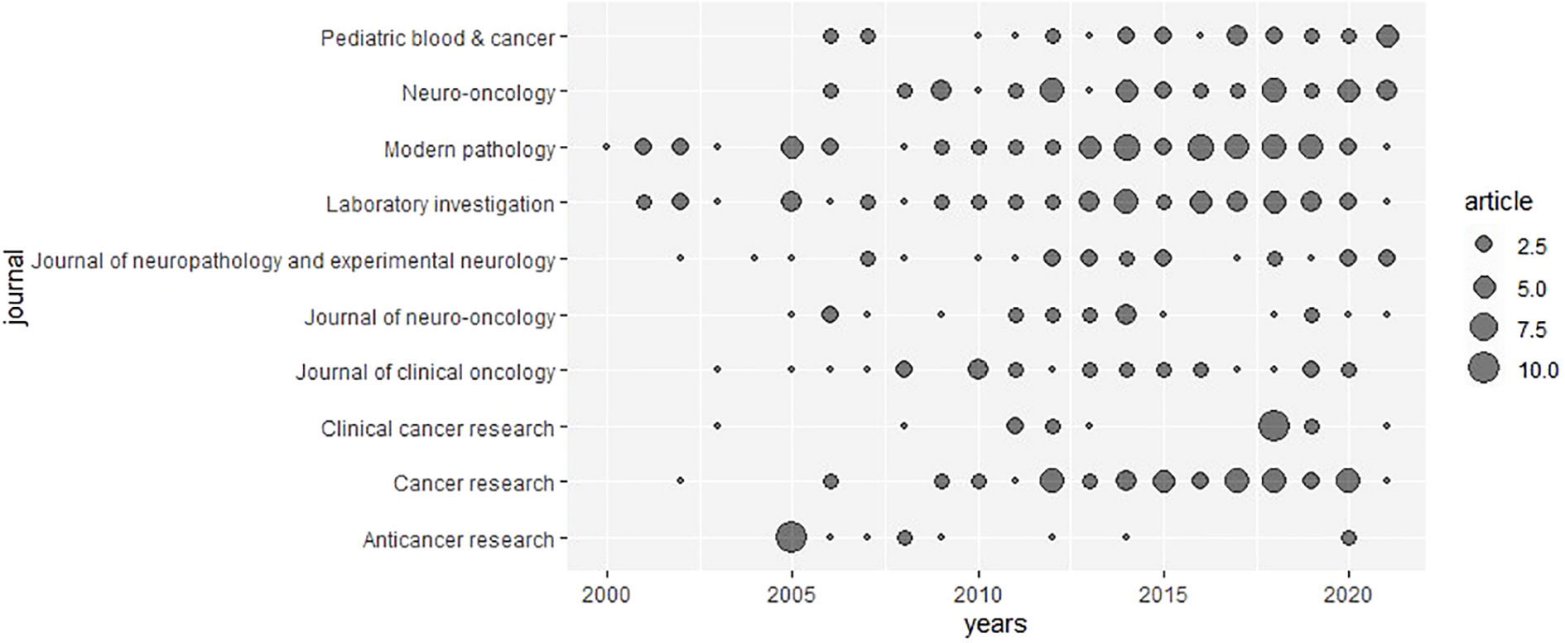
A visual representation of the chronological distribution of the publications from the top 10 most prolific journals.

A list of the top 10 MPNST journals, ranked by number of publications and total citations, is presented in **Table 6**. *Modern Pathology* ranked the highest, with 69 articles. Having published 56 articles, *Laboratory Investigation* was the second most popular journal, followed by *Cancer Research* with 50 articles. According to the 2021 JCR report, *Modern Pathology*, *Laboratory Investigation*, and *Cancer Research* are all included in journal impact factors (JIF) quartile Q1. Despite having an IF of 50.717 and being included in Q1, *Journal of Clinical Oncology* did not dominate MPNST research. As the most cited journal in MPNST research, *Cancer Research* recorded 822 citations with an H-index of 9. *The Journal of Medical Genetics* ranked second with only 4 articles, while the *American Journal of Surgical Pathology* ranked third with 749 citations and 14 articles. However, *Modern Pathology* had the lowest average citations, despite being the most popular journal. As illustrated in **Figure 7**, *Modern Pathology* held a dominant position during the investigated period.

**Table 6:** The top 10 journals based on publications and citations.

Rank	Journal	Publications	% Of 1400	IF(JCR 2021)	JIF quartile	Journal	Total citations	Publications	Average citations (AC)	H-Index	IF (JCR 2021)	JIF quartile	AC rank
1	Modern pathology	69	4.93	8.209	Q1	Cancer research	822	50	16.44	9	13.312	Q1	7
2	Laboratory investigation	56	4.00	5.50	Q1	Journal of medical genetics	777	4	194.25	2	5.941	Q1	2
3	Cancer research	50	3.57	13.312	Q1	American journal of surgical pathology	749	14	53.50	13	6.298	Q1	5
4	Neuro-oncology	47	3.36	13.029	Q1	Modern pathology	711	69	10.30	13	8.209	Q1	10
5	Pediatric blood & cancer	32	2.29	3.838	Q1	Nature genetics	569	3	189.67	3	41.307	Q1	3
6	Journal of clinical oncology	29	2.07	50.717	Q1	Clinical cancer research	549	21	26.14	9	13.801	Q1	6
7	Journal of neuropathology and experimental neurology	29	2.07	3.148	Q3	Neuro-oncology	540	47	11.49	13	13.029	Q1	9
8	Journal of neuro-oncology	22	1.57	4.506	Q2	PNAS	475	2	237.50	2	12.779	Q1	1
9	Clinical cancer research	21	1.50	13.801	Q1	Journal of clinical oncology	446	29	15.38	8	50.717	Q1	8
10	Anticancer research	19	1.36	2.21	Q4	Cancer	388	4	97.00	4	6.921	Q1	4

JIF, Journal Impact Factors

### 3.7 Highly cited articles

**Table 7** shows the top 10 most cited articles in the MPNST field, along with the top three cited articles.

**Table 7:** The ten most cited publications in MPNST.

RANK	First Authors	Citations	Article title	Journal abbreviation	Date	TC per Year	DOI	PMID
1	Evans DG	718	Malignant peripheral nerve sheath tumors in neurofibromatosis 1	J MED GENET	2002	34.19	10.1136/jmg.39.5.311	12011145
2	Berghmans S	446	tp53 mutant zebrafish develop malignant peripheral nerve sheath tumors	P NATL ACAD SCI USA	2005	24.78	10.1073/pnas.0406252102	15630097
3	Ferner RE	382	International consensus statement on malignant peripheral nerve sheath tumors in neurofibromatosis 1	CANCER RES	2002	18.19	NA	11894862
4	Lee W	308	PRC2 is recurrently inactivated through EED or SUZ12 loss in malignant peripheral nerve sheath tumors	NAT GENET	2014	34.22	10.1038/ng.3095	25240281
5	Anghileri M	248	Malignant peripheral nerve sheath tumors	CANCER-AM CANCER SOC	2006	14.59	10.1002/cncr.22098	16881077
6	Carli M	208	Pediatric Malignant Peripheral Nerve Sheath Tumor: The Italian and German Soft Tissue Sarcoma Cooperative Group	J CLIN ONCOL	2005	11.56	10.1200/JCO.2005.01.4886	16293873
7	De Raedt T	178	Elevated Risk for MPNST in NF1 Microdeletion Patients	AM J HUM GENET	2003	8.9	10.1086/374821	12660952
8	Stucky CC	175	Malignant Peripheral Nerve Sheath Tumors (MPNST): The Mayo Clinic Experience	ANN SURG ONCOL	2012	15.91	10.1245/s10434-011-1978-7	21861229
9	Zou C	172	Clinical, Pathological, and Molecular Variables Predictive of Malignant Peripheral Nerve Sheath Tumor Outcome	ANN SURG	2009	12.29	10.1097/SLA.0b013e3181a77e9a	19474676
10	Wasa J	170	MRI Features in the Differentiation of Malignant Peripheral Nerve Sheath Tumors and Neurofibromas	AM J ROENTGENOL	2010	13.08	10.2214/AJR.09.2724	20489098

1) “Malignant peripheral nerve sheath tumors in neurofibromatosis 1” (2002): There is a well-known association between MPNST and NF1. It has been reported that 1-2% of NF1 patients develop MPNST in cross-sectional studies, but population-based longitudinal studies that assess lifetime risk were before missing. Evans et al. first reported the 8–13% lifetime risk for NF1 patients of developing MPNST based on longitudinal studies. In addition, they found a worse prognosis in NF1-MPNST compared with sporadic MPNST (8).

2) “*tp53* mutant zebrafish develop malignant peripheral nerve sheath tumors” (2005): Berghmans et al. first reported that zebrafish lacking *tp53* spontaneously develop MPNST. Furthermore, they observed that animals with DNA damage had reduced apoptosis and cell-cycle arrest responses. The results indicate that *tp53* may play a tumor-suppressing role in the wild-type fish, and *tp53* mutation may contribute to MPNST tumorigenesis (9).

3) “International consensus statement on malignant peripheral nerve sheath tumors in neurofibromatosis” (2002): A consensus summary of MPNST in NF1 was presented by the multidisciplinary group for MPNST and NF1. They emphasized the importance of establishing an international database and standardized recording. They also noticed advances made with PET scanning and molecular genetics in MPNST. Moreover, multidisciplinary teams can bring benefits to MPNST management (10).

The top 10 most cited articles are mainly focused on molecular change in tumorigenesis and clinical management, which indicate that those topics were hot in MPNST research.

### 3.8 Most contributing institutions

**Figure 8:**
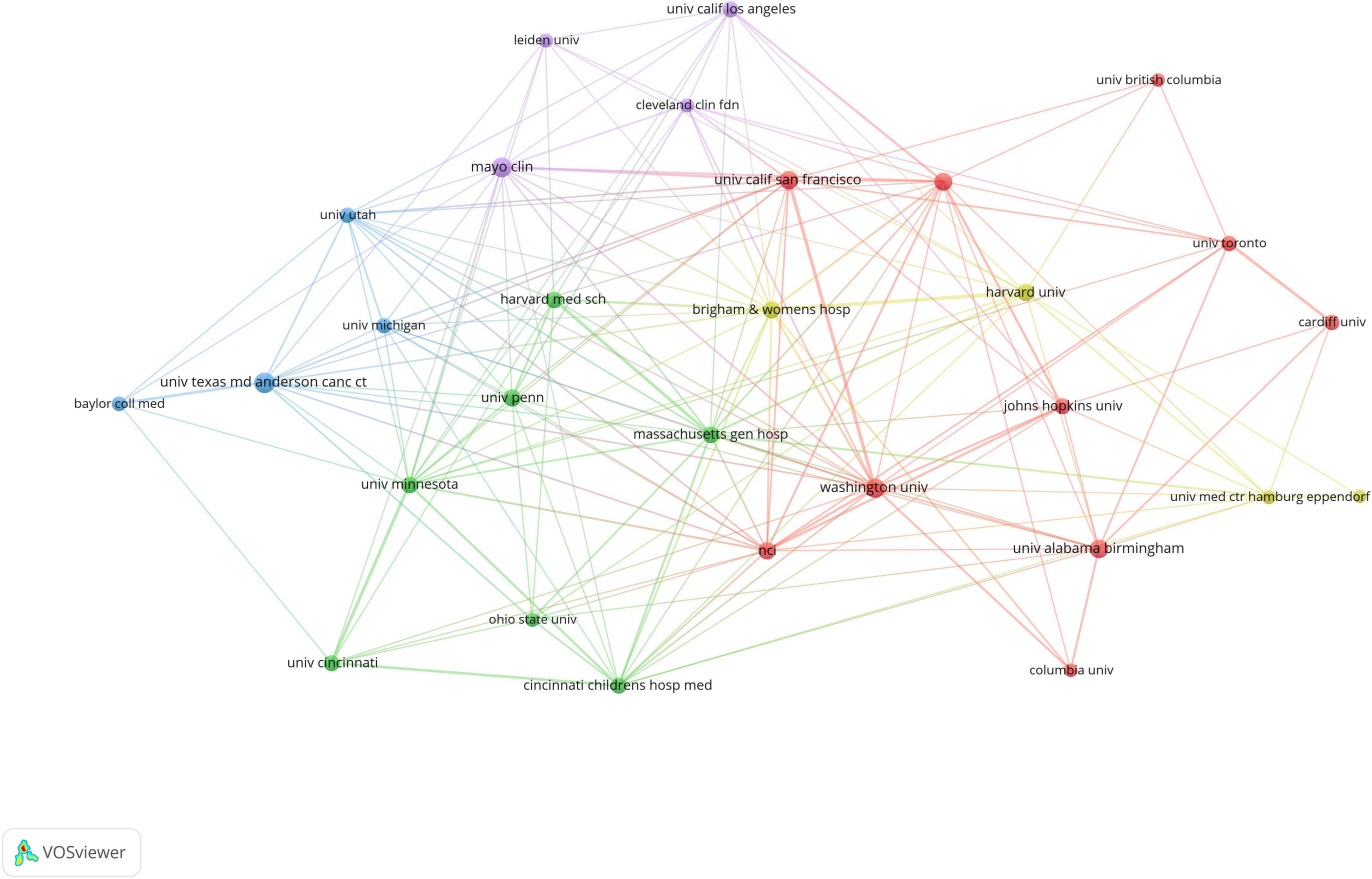
Visualization of collaborations between institutions VOSviewer.

The majority of the influential institutions are in the USA. The top ten most productive institutions are The University of Texas MD Anderson Cancer Center, Washington University, University of Alabama Birmingham, Mayo Clinic, Memorial Sloan Kettering Cancer Center, University of California-San Francisco, University of California-Los Angeles, Kyushu University, and University of Cincinnati. Despite publishing many articles, Kyushu University lacks strong cooperation in scientific research. The University of Texas MD Anderson Cancer Center is a significant collaborator in MPNST research (**Figure 8**).

## 4 Discussion

An analysis of bibliometric indicators indicated the research trends in this area and enabled the detection of changes, which will not only provide scientific guidance for future research work but also help us understand more about MPNST recently. The present study investigated 1400 publications from 2000 to 2022. As a relatively rare tumor, it is not surprising that few papers have been published on MPNST. However, inspiringly, the number of published articles has significantly increased. Compared to 2000, the number of articles published in 2021 is about five times higher. Consequently, the field of MPNST is currently gaining research attention. There was a downward trend in the average number of citations per article in MPNST, but this is not an accurate representation of article quality. Citations are more likely to increase with longer time after publication.

Among MPNST researchers, American authors dominate both article production and citations. However, Mautner (University Medical Center Hamburg-Eppendorf) was the author who published the most articles and received the most citations. What is more, he also led the H-index, G-index, and M-index. Clearly, his research has been highly productive as well as profound in its impact. Carroll and Perry are the second and third most productive authors. Among the top 10 high-impact authors, two are from University Medical Center Hamburg-Eppendorf, and two are from the University of California-San Francisco. Research in MPNST has been greatly enriched by those institutions.

With regards to number of publications, the USA dominates the list by a long way. In addition, they have the most experience in multi-country cooperation. Among the top 10 most productive countries, India and Korea are most likely to publish research by authors from a single country. It is likely that this multi-country collaborative research will become a trend and make a more significant contribution to MPNST research in the future.

Modern Pathology published 69 MPNST articles leading all other journals. *Cancer Research* received 877 citations and was the top-cited journal, while *PNAS* had the highest average citations per publication (237.50) with only two publications on MPNST. That is mainly attributed to the article titled “*tp53* mutant zebrafish develop malignant peripheral nerve sheath tumors” published in *PNAS* in 2005. This article was cited 446 times and has become the second most cited MPNST article since 2000. It is encouraging to note that by analyzing the productive and highly cited journals in MPNST, we found that most of the top 10 journals in MPNST are in the JIF Q1 division, including well-known and influential journals like *Nature Genetics* (IF: 41.307) and *Journal of Clinical Oncology*> (IF:50.717). As a result, much attention is being focused on MPNST-related research.

Compared to other bibliometric analyses in oncology (11–13), most of the research also analyzed the productive authors, relevant institutions, preferred journals, and high-citation articles to summarize the research trend. However, MPNST is a relatively rare tumor and a gain of knowledge from a small number of cases is limited. Institutions focused on the treatment of MPNST have the advantage of more extensive clinical data and biobanking. Cooperation with those outstanding institutions could be helpful. MPNST research led and influenced by these institutions will probably be the trend and will help to further discover the pathogenesis and predictive factors important for the prognosis in this rare disease.

## 5 Hotspots and frontiers

Using neutral conjunction of top keywords and literature, the following research hotspots were identified:

1) Tumorigenesis in MPNST: MPNST still lacks a specific treatment because its mechanisms are unclear. Thus, finding the trigger genes for MPNST tumorigenesis is vital for discovering new therapies. Berghmans et al. demonstrated in 2005 that mutant *tp53* zebrafish develop MPNST, and this paper became the second most cited publication in MPNST since 2000 (9). Lee et al. found PRC2 is recurrently inactivated through *EED* or *SUZ12* loss in MPNST (6). Patel discovered that inhibition of the *BET* bromodomain triggers apoptosis in MPNST (14).

2) Clinical management: De Raedt et al. observed that *NF1* microdeletion patients have an elevated risk of MPNST (2), and Tucker et al. reported a similar trend (15). Zou et al. analyzed 140 MPNST patients (72 were NF1-related, 68 were sporadic) and found MPNST ≥10 cm at diagnosis, partial resection, and metastasis development were significant negative predictors (16). Mautner et al. showed the difference in MRI between neurofibroma and MPNST (17). Warbey et al. indicated the value of [F-18]FDG PET/CT in diagnosing MPNST (18). Hagel et al. found patients were significantly younger at diagnosis (p < 0.001) and had a significantly shorter survival time than sporadic patients (19).

3) Predictive biomarkers: Zhang et al. performed genome-wide or targeted sequencing on 50 cases. Of the 50 cases, 16 MPNSTs but none of the neurofibromas tested were found to have somatic mutations in *SUZ12*, implicating it as having a central role in malignant transformation (20). Prieto-Granada et al. assessed 68 MPNST biospecimens and concluded that *H3K27me3* has good sensitivity and robust specificity for diagnosing MPNST (21). Endo et al. found that p-mTOR and p-S6RP were both indicators of poor prognosis (22).

## 6 Strengths and limitations

The bibliometric analysis helped improve understanding the research trends and hot spots in MPNST, and the visual presentation clarified the collaboration between authors and institutions. Moreover, our analysis of hotspot burst time and highly cited articles will provide further insights into the MPNST research and guide future research (**Figure 6**). Despite its strengths, the study does have some limitations. First, unless an article is included in the WOS database, it will not be included in this analysis. Since this analysis is based on the WOS database, more convenience is provided, but some of the detail was sacrificed in the process. Additionally, we searched for articles containing MPNST in the title, so some MPNST research publications might be overlooked if the term does not appear in their titles. The suggestion of cooperation institutions should not simplify ranked by the number of publications or citations, because each institution has respective interests, strengths and weaknesses. The recommendation needs to be judged upon the situation.

## 7 Conclusion

A steady increase in the number of publications on MPNST was observed from 2000 to 2022. The most influential author is Mautner (University Medical Center Hamburg-Eppendorf). The USA, Japan, and China were the three most productive countries. The journal *Modern Pathology* features the most MPNST publications, while those in the journal *Cancer Research* were the most frequently cited. The University of Texas MD Anderson Cancer Center may be a good partner to collaborate with. Recent research trends in MPNST have focused on tumorigenesis, clinical management, and predictive biomarkers.

## Data availability statement

The original contributions presented in the study are included in the article/supplementary material. Further inquiries can be directed to the corresponding authors.

## Author contributions

CG and XjH designed the experiments; XfH and ZF performed the Bibliometric Analysis; QG and SW check the data; JW and YS performed visualization in data; XfH wrote this manuscript. All authors contributed to the article and approved the submitted version.

## References

[1] Reilly K Kim A Blakely J Ferner R Gutmann D Legius E. Neurofibromatosis type 1-associated MPNST state of the science: Outlining a research agenda for the future. J Natl Cancer Inst (2017) 109(8). doi: 10.1093/jnci/djx124 PMC6057517 29117388

[2] De Raedt T Brems H Wolkenstein P Vidaud D Pilotti S Perrone F. Elevated risk for MPNST in NF1 microdeletion patients. Am J Hum Genet (2003) 72(5):1288–92. doi: 10.1086/374821 PMC1180281 12660952

[3] Nielsen GP Stemmer-Rachamimov AO Ino Y Moller MB Rosenberg AE Louis DN. Malignant transformation of neurofibromas in neurofibromatosis 1 is associated with CDKN2A/p16 inactivation. Am J Pathol (1999) 155(6):1879–84. doi: 10.1016/S0002-9440(10)65507-1 PMC1866954 10595918

[4] Stucky C-CH Johnson KN Gray RJ Pockaj BA Ocal IT Rose PS. Malignant peripheral nerve sheath tumors (MPNST): The Mayo clinic experience. Ann Surg Oncol (2011) 19(3):878–85. doi: 10.1245/s10434-011-1978-7 21861229

[5] Rhodes Steven D He Y Smith A Jiang L Lu Q Mund J. Cdkn2a (Arf) loss drives NF1-associated atypical neurofibroma and malignant transformation. Hum Mol Genet (2019) 28(16):2752–62. doi: 10.1093/hmg/ddz095. PMC6687955 31091306

[6] Lee W Teckie S Wiesner T Ran L Prieto Granada CN Lin M. PRC2 is recurrently inactivated through EED or SUZ12 loss in malignant peripheral nerve sheath tumors. Nat Genet (2014) 46(11):1227–32. doi: 10.1038/ng.3095 PMC4249650 25240281

[7] De Raedt T Beert E Pasmant E Luscan A Brems H Ortonne N. PRC2 loss amplifies ras-driven transcription and confers sensitivity to BRD4-based therapies. Nature (2014) 514(7521):247–51. doi: 10.1038/nature13561 25119042

[8] Evans D Baser M McGaughran J Sharif S Howard E Moran A. Malignant peripheral nerve sheath tumours in neurofibromatosis 1. J Med Genet (2002) 39(5):311–4. doi: 10.1136/jmg.39.5.311 PMC1735122 12011145

[9] Berghmans S Murphey R Wienholds E Neuberg D Kutok J Fletcher C. tp53 mutant zebrafish develop malignant peripheral nerve sheath tumors. Proc Natl Acad Sci USA (2005) 102(2):407–12. doi: 10.1073/p.nas.0406252102 PMC544293 15630097

[10] Ferner R Gutmann D. International consensus statement on malignant peripheral nerve sheath tumors in neurofibromatosis. Cancer Res (2002) 62(5):1573–7. 11894862

[11] Millagaha Gedara N Xu X DeLong R Aryal S Jaberi-Douraki M. Global trends in cancer nanotechnology: A qualitative scientific mapping using content-based and bibliometric features for machine learning text classification. Cancers (2021) 13(17). doi: 10.3390/cancers13174417 PMC8431703 34503227

[12] Zhang D Zhu W Guo J Chen W Gu X. Application of artificial intelligence in glioma researches: A bibliometric analysis. Front Oncol (2022) 12:978427. doi: 10.3389/fonc.2022.978427 36033537 PMC9403784

[13] Zhang Y Zhang X Wang X Han D Du J. Visual analysis of global research output of lymphedema based on bibliometrics. Front Oncol (2022) 12:926237. doi: 10.3389/fonc.2022.926237 35992843 PMC9389543

[14] Patel A Liao C Chen Z Liu C Wang Y Le L. BET bromodomain inhibition triggers apoptosis of NF1-associated malignant peripheral nerve sheath tumors through bim induction. Cell Rep (2014) 6(1):81–92. doi: 10.1016/j.celrep.2013.12.001 24373973 PMC3904298

[15] Tucker T Wolkenstein P Revuz J Zeller J Friedman J. Association between benign and malignant peripheral nerve sheath tumors in NF1. Neurology (2005) 65(2):205–11. doi: 10.1212/01.wnl.0000168830.79997.13 16043787

[16] Zou C Smith KD Liu J Lahat G Myers S Wang WL. Clinical, pathological, and molecular variables predictive of malignant peripheral nerve sheath tumor outcome. Ann Surg (2009) 249(6):1014–22. doi: 10.1097/SLA.0b013e3181a77e9a 19474676

[17] Mautner V Friedrich R von Deimling A Hagel C Korf B Knöfel M. Malignant peripheral nerve sheath tumours in neurofibromatosis type 1: MRI supports the diagnosis of malignant plexiform neurofibroma. Neuroradiology (2003) 45(9):618–25. doi: 10.1007/s00234-003-0964-6 12898075

[18] Warbey V Ferner R Dunn J Calonje E O’Doherty M. [18F]FDG PET/CT in the diagnosis of malignant peripheral nerve sheath tumours in neurofibromatosis type-1. Eur J Nucl Med Mol Imaging (2009) 36(5):751–7. doi: 10.1007/s00259-008-1038-0 19142634

[19] Hagel C Zils U Peiper M Kluwe L Gotthard S Friedrich R. Histopathology and clinical outcome of NF1-associated vs. sporadic malignant peripheral nerve sheath tumors. J neuro-oncol (2007) 82(2):187–92. doi: 10.1007/s11060-006-9266-2 17111191

[20] Zhang M Wang Y Jones S Sausen M McMahon K Sharma R. Somatic mutations of SUZ12 in malignant peripheral nerve sheath tumors. Nat Genet (2014) 46(11):1170–2. doi: 10.1038/ng.3116 PMC4383254 25305755

[21] Prieto-Granada C Wiesner T Messina J Jungbluth A Chi P Antonescu C. Loss of H3K27me3 expression is a highly sensitive marker for sporadic and radiation-induced MPNST. Am J Surg Pathol (2016) 40(4):479–89. doi: 10.1097/PAS.0000000000000564 PMC4882106 26645727

[22] Endo M Yamamoto H Setsu N Kohashi K Takahashi Y Ishii T. Prognostic significance of AKT/mTOR and MAPK pathways and antitumor effect of mTOR inhibitor in NF1-related and sporadic malignant peripheral nerve sheath tumors. Clin Cancer Res (2013) 19(2):450–61. doi: 10.1158/1078-0432.CCR-12-1067 23209032

